# Supplementary material for: Specific Secondary Bile Acids Control Chicken Necrotic Enteritis
Source: Pathogens. 2021 Aug 17;10(8):1041. doi: 10.3390/pathogens10081041 (PMC8427939; doi:10.3390/pathogens10081041)
Supplement: Supplementary file 1 [file pathogens-10-01041-s001.zip › pathogens-1328171-supplementary.pdf]

pathogens

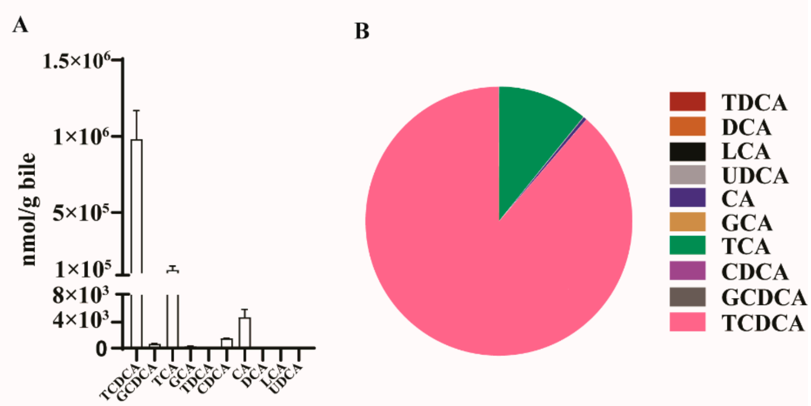

Figure S1. Bile acid composition of chicken bile. The chicken bile was collected aseptically from the gall bladder of broilers at day 56 in the processing plant of the University of Arkansas at Fayetteville. The bile acid composition of the chicken bile was determined by LC-MS/MS. (**A**) Quantitation of different bile acids. (**B**) Relative composition of bile acids.
